# Colonic Mucosal Ulceration and Gastrointestinal Bleeding Associated with Sevelamer Crystal Deposition in a Patient with End Stage Renal Disease

**DOI:** 10.1155/2018/4708068

**Published:** 2018-02-28

**Authors:** Sudheer Nambiar, Unnikrishnan Kunjan Pillai, Joe Devasahayam, Tony Oliver, Asha Karippot

**Affiliations:** ^1^Pulmonary & Critical Care Medicine, Cancer Treatment Centers of America, Tulsa, OK, USA; ^2^Nephrology, Indiana University, Muncie, IN, USA; ^3^Pulmonary Critical Care Medicine, University of Missouri, Columbia, MO, USA; ^4^USD Sanford School of Medicine, Vermillion, SD, USA; ^5^Hematology and Oncology, Cancer Treatment Centers of America, Tulsa, OK, USA

## Abstract

End stage renal disease (ESRD) population account for 1.9 per patient year of hospital admissions annually. ESRD population are at increased risk of bleeding secondary to use of anticoagulation during hemodialysis and uremia induced platelet dysfunction. Gastrointestinal bleeding accounts for 3–7% of all deaths in ESRD population. Lower gastrointestinal bleeding refers to blood loss from a site in the gastrointestinal tract distal to the ligament of Treitz. It is usually suspected when a patient complains of hematochezia. It is different from patients presenting with hematemesis that suggests bleeding from upper gastrointestinal tract. Common causes of lower gastrointestinal bleed include diverticulosis, ischemia, hemorrhoids, neoplasia, angiodysplasia, and inflammatory bowel disease. ESRD patients are known to retain phosphate alone or in combination with calcium which has been associated with high mortality. Sevelamer is a phosphate binder used widely in ESRD population. The known side effects of sevelamer include metabolic acidosis, vomiting, nausea, diarrhea, dyspepsia, abdominal pain, constipation, flatulence, fecal impaction, and skin rash. We are reporting a unique case of a 56-year-old female with end stage renal disease on sevelamer hydrochloride who presented with gastrointestinal bleeding and underwent a right hemicolectomy found to have sevelamer-induced mucosal ulceration and crystal deposition in the colonic mucosa. This case report highlights the fact that, with widespread use of this medication in the patients with chronic kidney diseases, physicians should be aware of this underrecognized entity in the differential diagnosis of gastrointestinal bleed in ESRD patients.

## 1. Introduction

In the United States, gastrointestinal bleeding is the most common cause of hospitalization due to a gastrointestinal disease. Approximately 30 to 40% of gastrointestinal bleeding is from lower gastrointestinal tract. Lower gastrointestinal bleeding refers to blood loss from a site in the gastrointestinal tract distal to the ligament of Treitz. It is usually suspected when a patient complains of hematochezia. It is different from patients presenting with hematemesis that suggests bleeding from upper gastrointestinal tract. Common causes of lower gastrointestinal bleed include diverticulosis, ischemia, hemorrhoids, neoplasia, angiodysplasia, and inflammatory bowel disease. End stage renal disease (ESRD) population account for 1.9 per patient year of hospital admissions annually. ESRD population are at increased risk of bleeding secondary to use of anticoagulation during hemodialysis and uremia induced platelet dysfunction. Gastrointestinal bleeding accounts for 3–7% of all deaths in ESRD population. ESRD patients are known to retain phosphate alone or in combination with calcium which has been associated with high mortality. Sevelamer is a phosphate binder used widely in ESRD population. The known side effects of sevelamer include metabolic acidosis, vomiting, nausea, diarrhea, dyspepsia, abdominal pain, constipation, flatulence, fecal impaction, and skin rash.

## 2. Case Presentation

**Figure 1:**
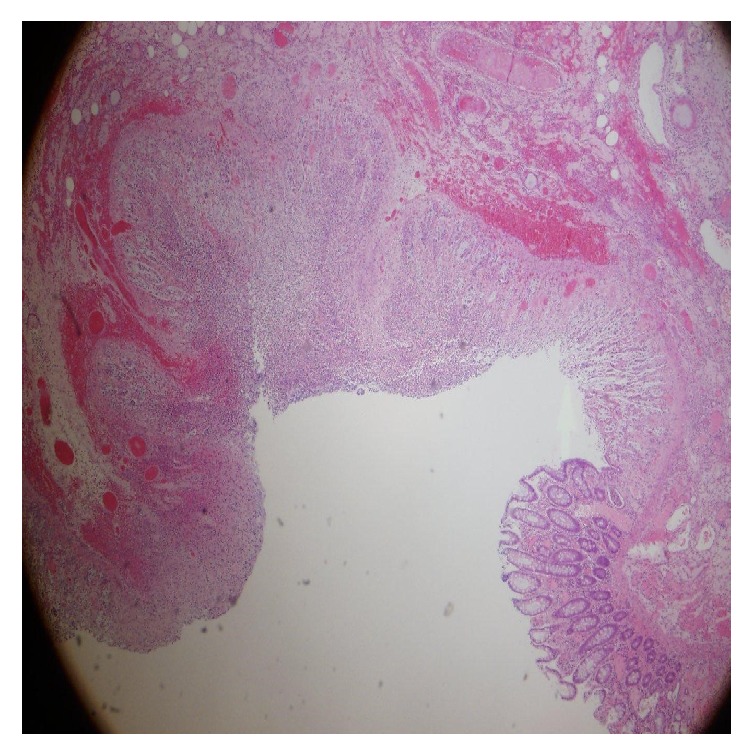
Mucosal ulcer with no atypical cells and crystals suggestive ulcer, inflammation, and gastrointestinal bleed (400x).

**Figure 2:**
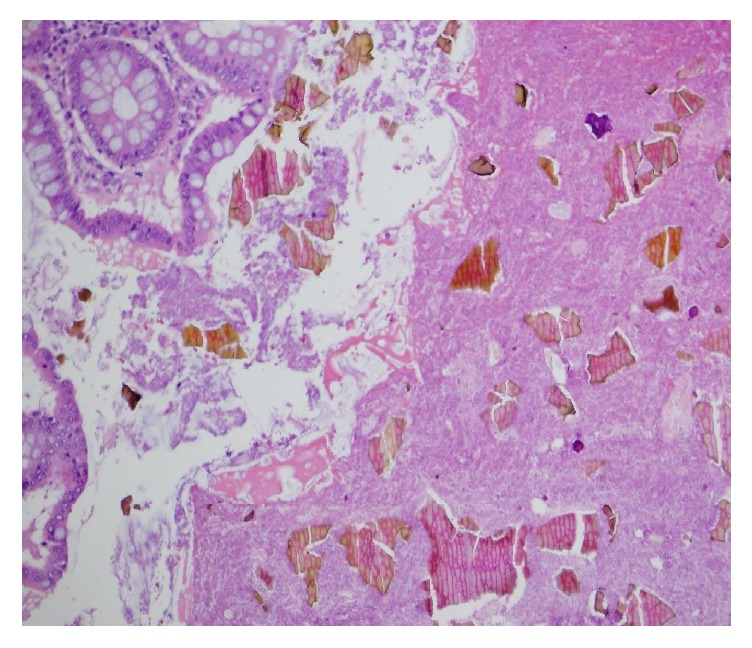
High power showing crystals deposition on hematoxylin and eosin stain (200x).

Here, we present a 56-year-old female with past medical history of hypertension, atrial fibrillation, ESRD secondary to hypertensive nephrosclerosis, being on hemodialysis three times a week for about four years, gastrointestinal reflux disease, and bronchial asthma who presented to the hospital with passage of bright red blood per rectum, syncope, and shortness of breath. Her home medications included amiodarone, aromasin, aspirin, carvedilol, diltiazem, pantoprazole, sevelamer hydrochloride, cinacalcet, and albuterol. The dose of sevelamer hydrochloride was 800 mg three times a day. Her clinical examination was unremarkable except for pallor. Orthostatic vital signs were not checked, as she was not complaining of dizziness. Her abdominal exam was benign. Her labs were remarkable for low hemoglobin at 5.7 mg/dl, serum sodium of 134 meq/l, and potassium of 5.5 meq/L, blood urea nitrogen was 46 mg/dl, serum creatinine was 8.5 mg/dl, serum calcium level was 10.3 mg/dL, serum phosphate level was 8.3 mg/dl, and PTH level was 555 pg/mL. Her INR was 1.1. No anticoagulants were given during the admission for deep vein thrombosis prophylaxis or during hemodialysis because of active gastrointestinal bleeding. The patient was given unfractionated Heparin during hemodialysis prior to the start of the gastrointestinal bleeding episodes. The anticoagulation protocol followed at that time was unfractionated Heparin bolus therapy (25–30 IU/Kg) at the beginning of the dialysis. After initial resuscitation with crystalloids, she was transfused two units of packed red blood cell. Gastroenterology consultation was made, and proton pump infusion was started. Upper gastrointestinal endoscopy was unremarkable; colonoscopy revealed erythema and ulceration near hepatic flexure with no active bleeding. A subsequent mesenteric angiography performed was normal. The next day, she underwent a bleeding scan following another episode of bleeding per rectum that showed activity near the hepatic flexure. She had to undergo an emergent right hemicolectomy for the continued bleeding. Postoperative period was uneventful. Patient bleeding stopped after surgery and hemoglobin stabilized. She was discharged from the hospital on the fifth postoperative day with appropriate follow-ups. Pathology report showed colonic mucosa with inflammation and ulceration, which was thought to be the site of bleeding (Figure 1). There were clusters of organophilic fish-like scale crystals, suggestive of sevelamer crystals that were noted in the colonic mucosa (Figure 2). Sevelamer was taken out of her medication list with close follow-up with her nephrologist.

## 3. Discussion

Treatment of hyperphosphatasemia is vital in chronic kidney disease (CKD) patients. Two methods used to treat hyperphosphatemia in nondialysis CKD patients are restricting dietary phosphate intake and administering phosphate binders to block absorption of ingested phosphate from the intestine. Sevelamer, a phosphate binder, is often used in treatment of hyperphosphatemia associated with chronic kidney diseases. Among 6797 patients enrolled in an observational, prospective study (Current Management of Secondary Hyperparathyroidism: A Multicenter Observational Study [COSMOS]), patients prescribed phosphate binders had a 29 percent lower risk of all-cause mortality and a 22 percent lower risk of cardiovascular mortality [1].

Sevelamer is well-known for its association with vascular calcification [2], endothelial damage, and increased mortality [3]. Phosphate binders, widely used to decrease oral phosphate absorption, are the mainstay of treatment. Sevelamer is a widely used phosphate binder, recommended by the Kidney Disease Improving Global Outcomes guidelines [4] in order to reduce the calcium positive imbalance in CKD patients. The known side effects of sevelamer include metabolic acidosis, vomiting, nausea, diarrhea, dyspepsia, abdominal pain, constipation, flatulence, fecal impaction, and skin rash. Sevelamer is associated with constipation in 8%–10% of the patients [5]. There has been a case report in which stercoral ulceration as a cause of lower gastrointestinal bleed has been ascribed to sevelamer use [6]. There were few case reports about sevelamer crystals in the gastrointestinal tract. The common sites of involvement included the esophagus, small bowel, and colon of that intestine which was involved in 81% of the cases [7]. Notable mucosal abnormality included chronic mucosal damage, acute inflammation, inflammatory polyp, extensive ulceration, ischemia, and necrosis. Sevelamer crystals displayed broad, curved, and irregularly spaced “fish scales” or “tree bark” appearance with a variably eosinophilic to rusty brown color on hematoxylin and eosin staining and violet color on periodic acid-Schiff-alcian special staining with diastase [8]. Diabetics seem more prone to develop sevelamer crystals associated gastrointestinal lesions [7]. In our case report, we cannot exclude other possible or enhancing causes of gastrointestinal injuries. A coincidental sevelamer deposition on a previously injured area is a possibility, but the foreign body reaction observed around the area strongly suggests causality. Most cases of sevelamer crystals are benign and are found in incidental pathology specimen; few patients present with life-threatening gastrointestinal bleeding as in our case.

## 4. Conclusion

Several studies exist regarding the causes of gastrointestinal bleeding in ESRD patients. To our knowledge, there is only a handful of literature on the sevelamer crystals identified in colon presenting with gastrointestinal inflammation, ulceration, and bleeding. This side effect of sevelamer remains unrecognized by many nephrologists and practitioners. This case highlights the fact that, with widespread use of this medication in the patients with chronic kidney diseases, physicians should be aware of this entity.

## Nonstandard Abbreviations

ESRD: End stage renal disease
CKD: Chronic kidney disease
INR: International normalized ratio.
